# Making New Health Services Work: Nurse Leaders as Facilitators of Service Development in Rural Emergency Services

**DOI:** 10.3390/healthcare6040128

**Published:** 2018-10-27

**Authors:** Helle Kise Hjertstrøm, Aud Obstfelder, Bente Norbye

**Affiliations:** 1 Department of Health and Care Sciences, Faculty of Health Sciences, UiT The Arctic University of Norway, 9019 Tromsø, Norway; bente.norbye@uit.no; 2 Department of Health Sciences in Gjøvik, NTNU, Faculty of Medicine and Health Sciences, Norwegian University of Science and Technology, 2802 Gjøvik, Norway; aud.obstfelder@ntnu.no

**Keywords:** nurse leaders, rural nursing, organizational work, articulation work, qualitative research, service development, health policy, rural emergency clinic

## Abstract

Nurse leaders in middle management positions in Norway and other Western countries perform additional new tasks due to high demands for quality and efficacy in healthcare services. These nurses are increasingly becoming responsible for service development and innovation in addition to their traditional leadership and management roles. This article analyses two Norwegian nurse leaders efforts in developing an emergency service in rural municipal healthcare. The analysis applies an ethnographic approach to the data collection by combining interviews with the nurse leaders with observations and interviews with six nurses in the emergency service. The primary theoretical concepts used to support the analysis include “organizing work” and “articulation work”. The results show that in the development of an existing emergency room service, the nurse leaders drew upon their experience as clinical nurses and leaders in various middle management positions in rural community healthcare. Due to their local knowledge and experience, the nurses were able to mobilize and facilitate cooperation among relevant actors in the community and negotiate for resources required for emergency medical equipment, professional development, and staffing to perform emergency care within the rural healthcare context. Due to their distinctive professional and organizational competency and experience, the nurse leaders were well equipped to play a key role in developing services. While mobilizing actors and negotiating for resources, the nurses creatively balanced these two aspects of nursing work to develop the service in accordance to their expectation of providing the highest quality of nursing care to their patients. The nurse leaders balanced their professional ambitions for the service with legal directives, economic incentives, and budgets. Throughout the development process, the nurses carefully combined value-based and goal-based management concerns. In contrast, other studies investigating nursing management and leadership have described that these orientations are in opposition to each other. This study shows that nurses leading the processes of change in rural communities manage the change process by combining the professional and organizational domains of the services.

## 1. Introduction

Many European countries face challenges in the delivery and organization of healthcare, leading to increased demands for service development and innovation [1]. However, service development and innovation are challenging. One way of promoting success is by engaging professionals in management roles at ward levels to actively work towards service development and innovation [2,3,4]. Thus, nurse leaders are no longer solely in charge of daily operations, budgeting, and ensuring the professional quality of the delivered healthcare; they are also increasingly responsible for service development and implementation [1,5]. Thus, these nurses manoeuvre complex dynamic healthcare systems [6,7] by applying both professional and organizational knowledge to the delivery and organization of their and other professionals’ work [8]. Because of this particular experience, nurse leaders are considered important actors in the development of new services and the implementation of innovations [9]. However, few studies have investigated how nurse leaders perform service development and innovation and what competencies this requires.

This article explores how two nurse leaders facilitated the development of a new politically initiated health service in a rural context of municipal emergency wards and what competencies guided their work. Through this new general medical service, the municipals are obligated to offer 24-h emergency services to patients in need of immediate help and observation as an alternative to hospitalization [10]. The Norwegian Directorate of Health has recommended integrating the emergency observation bed service model into existing health services [10] as a part of the expansion of the responsibility for acute patients to municipal healthcare [11].

The methods used to gain insight into how the nurse leaders accomplished their responsibilities in the development of the new service were qualitative. The data were collected by observing and interviewing the nurse leaders regarding their work related to service implementation. Nurses working at the emergency clinic were also observed and interviewed. In our data analysis, we drew on the theory of the social organization of healthcare work, particularly the concepts “organizing work” and “articulation work” [12,13,14]. This theoretical perspective allowed us to view healthcare as a complex social organization in which professional knowledge and values, regulative and managerial structures and incentives were integrated into the organizational structures [15,16]. These structures continuously shape and are shaped by the daily work of professionals. This article shows that the nurse leaders facilitated the development of the emergency ward by negotiating for new forms of organizational orders. In their negotiations of the service that should be delivered and organized, the nurses linked the formal organizational structures to professional values in the development of the municipal emergency beds.

### 1.1. Background

The Norwegian healthcare system is divided into two levels: specialist and municipal health services. Specialist health services include public hospitals and ambulance services and are state governed. Municipal health services are primary services that include emergency care, general practice (GP), home-based care, and nursing homes.

The most recent comprehensive health reform in Norway, i.e., the Coordination Reform, occurred in 2012 [10]. This reform aimed to achieve better and more effective healthcare for individual patients by transferring the responsibility for service delivery to municipalities and improving collaboration and cooperation between specialist and municipal services and within municipal services [10].

In rural municipalities, the new requirements for quality and competences set by the reform are challenging to meet [17]. However, one of the main actions of the Coordination Reform has been to establish municipal emergency wards. The Norwegian Ministry of Health and Care Services has provided guidelines for the organization of these services, but municipalities are allowed to adapt these services to local contexts [18]. Thus, emergency wards vary in terms of their size and services offered [11].

Regarding the financial incentives to establish the new service, municipalities could apply for earmarked grants from the Norwegian Directorate of Health and funding from cooperating health trusts between 2012 and 2015 [10]. Since 2016, all funding is incorporated into the government block grants to municipalities. The new service is restricted to emergency admissions with the possibility of observation and treatment for up to 48 h. To avoid exceeding the 48-h period, the health authorities recommend that municipalities enhance competencies and capacity in alternative services, such as nursing homes and home care, in parallel to developing the new emergency service [18]. Furthermore, municipal emergency clinics and associated ambulance services, emergency medical communication centres and emergency hospitals must adapt to the changes in expertise and emergency preparedness involved in the new service. Therefore, the health authorities require municipalities and emergency hospitals to draw up legally binding cooperation agreements regarding patients offered the new service. These agreements should include descriptions of the criteria for using the service [18].

For the service to operate effectively, the health authorities recommend that municipalities plan and describe how they aim to establish a staff of competent professionals who will make correct decisions regarding whether patients should be admitted for observation or referred to a hospital [19]. These plans and descriptions should also include access to necessary technical medical equipment, documentation systems and other support functions as well as systems of internal controls. There should also be plans and descriptions of the development of expertise in observation, assessment and action. Since admission to the ward is based on observations of the patient’s general condition, the severity of the emergency condition, and psychosocial factors, such expertise is essential for the sound operation of the service. Other factors involved include the development of solid skills in medical procedures, such as cardiopulmonary resuscitation; the use of equipment, such as an ECG monitor and pulse oximeter; oxygen treatment; blood tests; catheterization; and IV fluid and medication administration [18,19].

### 1.2. Context

The initiative for developing the municipal emergency ward, which is the focus for this study and occurred in 2011, was one of the first initiatives to receive national funding. Currently, the initiative consists of two emergency beds implemented in an intermunicipal medical clinic in northern Norway. The medical clinic includes an emergency room service. The intermunicipal collaboration at the emergency room service involves four municipalities covering a large area with two to three hours of travel time to the nearest hospital. Occasionally, due to rough winter conditions, road access is closed. The emergency room service is staffed by two nurses who are on call 24 h, a doctor on call outside of office hours and emergency ward doctor available during the day. Both the emergency ward doctor and on-call doctors at the emergency clinic are also employed as local GPs. The nurses are responsible for monitoring and caring for the patients admitted to the emergency beds, operating the rural emergency room communication centre, and caring for the patients admitted to the emergency room clinic for medical examination.

The nurse leaders who were key informants in this study were given the responsibility of planning and developing the emergency beds from their municipal leaders for the following three reasons:

First, when the health authorities directed the Norwegian municipalities to expand their emergency services from emergency room clinics to municipal emergency ward clinics with bed facilities for acute and critical care observation, the nurse leaders were already involved in a related initiative at the local emergency room clinic. The health authorities recommended that the new emergency service should be established within related locations. It was planned for the new emergency acute beds to be located on the premises of the local emergency room clinic.

Second, the local emergency room clinic was a part of an intermunicipal medical clinic, and the two nurse leaders had been involved in implementing this clinic. The intermunicipal emergency room clinic was initiated by four neighbouring municipalities in 2007 to enhance the quality and capacity of emergency care and recruit and retain medical doctors and other healthcare professionals to the region.

Third, the nursing staff at the emergency room clinic were more affected by the changes than the medical staff, and when the authorities proposed the new service, the doctors were not very engaged in preparing for the upcoming changes. The new service implied that the nurses should provide nursing care for patients at a high risk of potentially life-threatening health problems. As the emergency room nurses had little experience with this type of “intense and vigilant” nursing care, initiatives that ensured the highest level of nursing care for these patients were crucial.

### 1.3. Theory

Individual and organizational change is a central theme in studies investigating health service management [13,14,15,20,21]. Studies underpinned by the institutional logic perspective present the activities and preferences of professionals as situated forms of organization that are linked to the beliefs and practices of wider institutional environments [22]. These beliefs and practices are “institutional logics” that have the capacity to influence their orientation to work and also provide an opportunity for agency and change. While professionals may seek autonomy in work performance, the means and ends of their aspirations and related activities are both enabled and constrained by prevailing institutional logics [15,22].

In these studies, organizational logic and professional logic are usually described as being diametrically opposed. Organizational logic is related to the structures and management of healthcare services, and professional logic involves the values of the services [23]. Goal-oriented management (to achieve effective services) is considered to be in contrast to value-based management (to provide professional care for patients) [24]. In research on goal-oriented management, professionals are often presented as stressed and demotivated due to higher performance management pressure, and managers are presented as failing to understand professionals’ passion about their patients, which they consider a barrier to change.

This dichotomy does not reflect our observations of the nurse leaders work. In their interviews about their responsibilities in developing the municipal emergency beds, the nurse leaders did not clearly separate the organizational and managerial aspects of their work from their patient-oriented work. Furthermore, the nurses did not discuss how the characteristics of the rural area, such as distance to the nearest city, climate, and accessibility to healthcare professionals and medical expertise influenced their work. Nevertheless, even if new studies note the interaction between these logics [14,15] and how the duties and tasks associated with specific professions develop in response to societal and organizational changes [25], the work of nurses related to organizational and management of care is still often presented in parallel to bed-side care and direct interaction with individual patients, which is considered true professional work [26]. The work environments of nurses are usually portrayed as external to patient-centred work, this is limiting for how the work of nurses is understood [13].

To extend beyond these dichotomies and explore how the two nurse leaders aligned elements from each dichotomy in their effort to develop the municipal emergency care beds, we refer to Davina Allen’s study of nurses’ organizing work [13]. The concepts “organizing work” and “articulation work” are particularly relevant to our analysis. According to Allen [13], healthcare is an evolving activity accomplished under conditions of complexity. Patients receive input from different providers and specialists, and these relationships are conditioned by differences in knowledge, occupational cultures, socials worlds, power and prestige. Daily service provision is characterized by action and knowledge that is distributed across time and space, and the fragmented and multiple understandings of patients and staff provide largely independent contributions to care. High-quality healthcare depends on ensuring that necessary information, people and materials meeting individual patients’ needs are aligned [27]. The concepts ‘organizing work’ and ‘articulation work’ capture the role of nurses in aligning these elements.

While organizing work more generally refers to the ordering work of nurses and its evolving and continuous character, “articulation work” refers to particular practices that assemble and align the elements. Ordering work involves an understanding of and skilled negotiation for relationships among the elements that need to be aligned to ensure care, i.e., the patients and their social network and different organizations, departments, professions and occupations. While the overall aims of patient care may be shared, the parties involved often have particular concerns, reflecting their respective roles and responsibilities. Articulation work is required for the work to be effective [28,29]. The complex and distributed characteristics of healthcare require that decisions are made about what should be done, by whom, where and with what materials. Care is uncertain, and emergent situations and activities related to individual patient care trajectories cannot be fully planned in advance. Despite formal structures and processes, large aspects of healthcare work are undertaken in parallel to those involved and are distributed across departments and specialities. However, nurses combine their awareness of individual patient trajectories with an understanding of organizational processes to undertake articulation work to ensure that events occur at the right time in the right order, i.e., the availability of recourses and material to support action and the coherence in and between patient trajectories [27].

In the analysis of the nurse leaders work, the analytical power of these concepts is utilized.

Cooperation among different actors is a prerequisite for an efficient workflow and work organization. Cooperation does not occur automatically in a vacuum. In contrast, it is the result of ongoing negotiations, adaptations and adjustments in activities and responsibilities across time and space. The development of new services inevitably involves the re-ordering of established patterns of cooperation and negotiation. Activities and responsibilities in and across dimensions of time and space must be aligned in new and likely unexpected ways.

## 2. Material and Methods

Similar to previous studies performed in the hospital environment, we chose an ethnographic approach [13,30,31]. We aimed to obtain insight into the organizational setting in which the emergency ward was developed and knowledge regarding health care personnel’s daily work. Thus, we aimed to understand why and how nurses acted as they did.

To provide an overview of the field, the authors held a meeting with the two nurse leaders, who discussed their daily work and their development and facilitation efforts for the new service. Notes from this meeting were used as the basis for in-depth interviews with the nurse leaders. We also conducted in-depth interviews with six nurses. To enhance our understanding of the development of their practice, their daily work at the emergency ward clinic (250 h) was observed in the field by the first author. This fieldwork was conducted periodically by the first author (H.K.H.) from September 2014 to June 2015. Because of the nature of the work and patients were admitted with an immediate need for treatment and care, shadowing was used throughout the fieldwork period [32]. This process partially involved in-situ interviews with the nurses and nurse leaders, which provided deeper insight into the observations by confirming or expanding specific events [33]. The in-situ interviews were often based on specific observations. The nurse leaders were asked to describe procedures, elaborate upon situations and explain their actions, which enhanced our understanding of their daily work and corrected misinterpretations.

### 2.1. Analysis

The data analysis began during the first meeting with the nurse leaders and continued throughout the fieldwork. H.K.H. constantly checked her observations, interpretations and understandings against those of the informants. We obtained the informants’ understanding of how their professional activities and actions were shaped by organizational factors. Therefore, we asked not only about events that occurred but also the determinants of these events [33]. In the analysis, we incorporated written manuals, registration forms and guidelines. The nurses were asked how these texts were used and why. Then, we performed a thematic analysis [34] and used the qualitative analysis software NVivo (QSR International Pty Ltd. Version 10, Doncaster, Australia) to sort and code the data. This article addresses the following questions: In what way do nurse leaders facilitate the development of municipal emergency beds? What competencies underlie their work? We designed our research questions according to our analytical framework and searched for patterns in the nurse leaders stories and descriptions of their work in the rich data obtained from these observations and interviews. This analysis shed light on the dynamics between the social organization of the emergency beds and the simultaneous reshaping of the clinical practice at the emergency ward clinic. As a result of this analytical process, three aspects of the dynamics were identified and are presented below.

### 2.2. Ethical Considerations

This study was approved by the Norwegian Centre for Research Data (reference: 3889) and the Regional Committees for Medical and Health Research (reference: 2014/1658). Anonymity and confidentiality were assured according to standard procedures.

## 3. Results

These results describe how the nurse leaders actively got involved in and contributed to developing the municipal emergency ward. The results are presented as three different aspects of organizing work and articulation work, reflecting the theoretical framework upon which the analysis was based.

### 3.1. Facilitating Trust and Resource Mobilization

Facilitating trust is related to how the nurse leaders mobilized key actors to develop and adapt the services to their context. The nurse leaders build on their previous experiences to establish the rural emergency care service, and they closely cooperate with different health professionals in the region. Mobilizing GPs was particularly important, and the nurse leaders knew the GPs from previous service development initiative. GPs are responsible for admitting patients to the emergency beds and have the juridical responsibility of providing medical treatment to the population in their municipalities [11,18]. The nurse leaders emphasized that the development of the emergency ward led to a new type of interaction with GPs as follows:

“We also cooperated with different GPs in surgeries before. We cooperated on referrals or discharge summaries […] and what is new about the emergency beds is that we cooperate on a specific service. The GPs admit patients from their surgeries, but the patients are here with us. It is the nurses that look after the patients. A GP has to attend and check on the patient and respond when we call for support”. In situ interview quote, nurse leaders.

The emergency regulations require continuous 24-h nurse presence to observe and care for patients [18]. Because the GPs are legally responsible for the patients they refer to the emergency beds, the nurse leaders were concerned about the GPs’ willingness to trust the nurses’ professional skills. Indeed, due to the distance to the nearest hospital and the Arctic climate, the GPs were highly dependent on the nurses having the right skills and capacity to care for the patients in a qualified manner. The negotiation with the political management of the emergency clinic to increase the number of nurses during each shift was central to the nurse leaders attempt to motivate the GPs to participate in the establishment of the emergency beds. The nurse leaders expressed a clear perception that a professional rural emergency clinic with emergency beds required two nurses per shift. However, this requirement was not the case before the emergency beds were implemented. The nurse leaders indicated that operating the local emergency medical communication centre, following up with patients arriving for referrals and caring for patients admitted for observation and treatment in an emergency bed would be impossible with only one nurse on duty. Thus, another trust-building measure was undertaken to inform the GPs about the emergency ward while emphasizing the extension of the nursing capacity at the emergency clinic. This measure was performed in response to the GPs’ input regarding the requirements for referring patients to the emergency beds:

“For good cooperation, GPs must have confidence in the emergency ward and recognize our skills. We are spending a considerable amount of time informing the GPs about the emergency ward, encouraging dialogue between them and us, and motivating them to use it. It is time-consuming and challenging but necessary”. Observation note; quote from meeting between nurse leaders.

The nurse leaders actively worked to motivate the GPs to engage in the development of the emergency ward. According to the nurse leaders, this motivational work was also important for the process of developing new work relations and collaboration between the GPs and nurses. The nurse leaders believed that this process could lead to mutual trust, better communication, and consequently, better quality of the emergency services.

### 3.2. Facilitating Procedures and Cooperation

The second aspect of the dynamics between the social organization of the emergency beds and the reshaping of the clinical practice at the rural emergency clinic involved the nurse leaders effort to develop procedures for the observations and care for the admitted patients. Patient treatment and care should be secured and prevent a long travel to a hospital if the patient’s condition could be treated at the rural emergency clinic. According to the nurse leaders, the procedures included the structuring of activities, such as continuous observations and evaluations of patient status, including cooperation between different professions and GP practices, and the division of responsibilities. Local knowledge regarding available professional expertise and the distance to other health services were included in these procedures. The procedures were designed to ensure the quality of care for patients by specifying tasks, activities and responsibilities.

The development of the procedures involved discussions among the nurse leaders, the GPs and other healthcare services in the municipalities about shared responsibilities and collaboration regarding the patients admitted to the emergency beds. The nurses sought to communicate with the GPs regarding admissions to the emergency beds and other service providers, such as homecare nurses and nursing home staff, regarding follow-up care. Because the municipal emergency ward formed a part of the intermunicipal collaboration, care activities for some patients had to be coordinated across the four neighbouring municipalities. To facilitate this coordination, the nurse leaders initiated procedures that gave them an overview of all tasks and responsibilities and clearly defined responsibilities among the different professions and service providers. One nurse leader provided the following example of how complex the cooperation for patient care might be:

“We’ve had varying collaboration on patients across the various municipal health services since we started the intermunicipal rural emergency clinic. The emergency beds increase the need for cooperation. For example, a typical acute patient may have heart failure and sometimes need more treatment than home-based nursing or the GP can provide. If the patient’s condition deteriorates while being observed at the rural emergency clinic, the nurses must contact the GPs or emergency clinic doctor if the GPs are not on duty. The GPs must consult with the hospital doctor, and the nurse must prepare to request an ambulance and make sure the patient record is included. Alternatively, if the patient’s condition stabilizes and they can be discharged from the emergency beds, then a nursing home or the GP should be contacted to clarify further treatment. Perhaps the homecare nurse should also be contacted”. Interview, nurse leader.

The nurse leaders were aware that the emergency bed service could compete with the nurse’s attention and that the quality of their ordinary work at the rural emergency clinic could decrease. Therefore, the nurses wanted to improve their competence while they implemented the emergency beds as a service in the emergency clinic. However, although the Norwegian Directorate of Health intended for the municipal emergency ward to be a full-fledged alternative to hospitalization [18], the nurse leaders said that this goal was difficult to put into practice immediately. Based on their clinical experiences as nurses, the nurse leaders foresaw how the requirement of nursing competence for the observation and care of emergency bed patients differed from their previous practice:

“We’re used to all types of patients in our region. What’s new about the municipal emergency ward is that we now have to observe and treat some of them here at the clinic. We have developed good procedures for the emergency clinic and taking emergency calls, and now, we want the emergency ward patients to have good care too. The comfort and safety of both the patients and staff are important, and we have to develop procedures that ensure this. The procedures should reflect our work here, but we do not quite know what the emergency ward would be like, and we have to try things out. We included clinical nurses in the development of the procedures and tried to include doctors too. The procedures must be suitably clear and flexible. Of course, they must be revised several times and are being developed, but we have performed a thorough job”. In-situ interview quote, nurse leader.

The nurse leaders had an influence on the development of the emergency beds in the clinic by developing procedures for care, and they foresaw how the emergency beds could lead to changes in the nurses’ work. According to the nurse leaders, the nurses needed not only professional skills for operating the local emergency medical communication centre but also skills for the observation and care of critically ill patients. If a patient’s condition deteriorated, the nurses had to prepare for timely travel by ambulance or helicopter to secure the patient for transport. Because the nurse leaders had previous experience as nurses in clinical work, they understood how the changes could affect the nurses’ work practices and the need for updated competence. In planning the new service, including how this concern could be integrated into the emergency clinic, the nurse leaders relied on previous experience.

### 3.3. Facilitating Adjustment of Professional Competence

The third aspect of the dynamics between the social organization of emergency beds and the reshaping of clinical practice at the rural emergency clinic was related to how the nurse leaders managed the *process of adjusting professional competence*. As shown above, the emergency beds changed the duties of both the nurses and GPs.

The nurse leaders previous experience from establishing the emergency clinic and the intermunicipal collaboration prepared the nurses for periods of uncertainty and disagreement among the professionals involved. The nurse leaders wished for a smooth process to avoid unnecessary conflict and therefore included all relevant professionals in the process while also being responsive to and prepared for disagreements:

“We know from experience that it takes a couple of years to get things sorted out. We doubled the staff, instituted new procedures, and offered a brand-new service. We were prepared for a period of uneasiness, turnover, worried staff and disagreements. We’ve seen it before, and we saw it with the emergency ward […] It’s important to have a dialogue with the staff—they must feel they own it all. It’s especially important with nurses, they’re the continuity here. They must feel that we’re listening to their input. They must feel sure about the emergency ward”. Interview, nurse leader.

Although the municipal emergency ward is primarily written as a GP service in the national regulations [18,19], the nurse leaders realized that the nurses could experience the greatest changes in practice. As shown above, the nurse leaders realized the importance of ensuring that two nurses were on duty and developing good procedures, and they were also concerned about creating a safe working framework for the nurses in the clinic. The nurse leaders were familiar with the clinical work at the emergency clinic; consequently, they were concerned about allowing time for the nurses to operate the rural emergency medical communication centre and follow up on the patients at the emergency clinic. In addition, the work extended to learning to monitor and care for critically ill patients over time. However, although the nurse leaders were confident that the emergency clinic had sufficiently qualified staff to care for patients in need of continuous monitoring and close follow-up, certain nurses were less confident:

“As leaders, we feel sure about the emergency beds. The expertise is here, and we know it will improve work in the emergency clinic over time because the foundation’s good. We’ve tried and failed and have lots of experience we use actively. Both we and the nurses have this. We’re keen to boost the nurses’ confidence and calm them down. That takes time”. Interview, nurse leader.

The nurse leaders expected uncertainty among the staff and foresaw how the emergency beds could affect the established practices at the emergency clinic more than the policy guidelines suggested. The distance to hospitals had to be considered, and the few colleagues available influenced the responsibility experienced by each nurse. Additionally, in this aspect, the nurse leaders actively used their experience from previous positions in planning how the development of the acute bed service would unfold.

## 4. Discussion

“There are many simultaneous processes and many needs to be constantly addressed—nurses, doctors, intermunicipal collaboration, Directorate of Health, and the local government. You name it. Most important of all is, of course, the patient. All the time, we’ve worked to provide good patient care; we’ve been thinking professionalism and quality all the way. There’s a lot of organization behind good patient work. That is important”. In-situ interview quote, nurse leader.

This study explored how nurse leaders in rural municipalities facilitated the development and establishment of a municipal emergency ward and the knowledge and skills that supported this contribution. The quotation above illustrates our findings. The nurse leaders actively used their knowledge and experiences, both clinical and administrative, as a body of knowledge in this process. This knowledge and experience are expressed in their ability to negotiate and facilitate collaboration and trust among colleagues as they developed routines based on professional standards that ensured compliance with both the outpatient emergency service and the included emergency beds. Their knowledge of this lived place [35], including its geography, distance to hospitals, and changeable weather that might prohibit helicopter transport, was a part of their capacity.

Consistent with Postma et al. [26], the organizational and professional aspects were observed to be integrated into the nursing practice. The analysis shows that the nurse leaders do not necessarily consider coordination and planning around the service development distinct from professional knowledge and values. This aspect of integrated knowledge is consistent with Noordegraaf [14] and Bévort and Suddaby [24]. This finding illustrates the nurse leaders dilemma when nurses must simultaneously operate the rural emergency medical communication centre, follow up on emergency clinic patients and monitor and care for the patients in the emergency beds. These complex health services were the responsibility of two nurses per shift. At the communication centre, the nurses triaged patients via phone and decided whether they needed medical attention or it was sufficient to advise them on how to address the problem at home. Following-up on the patients at the clinic involved close cooperation with the GP on call. The patients in the two emergency beds had to be carefully monitored for any change in condition. The nurse leaders developed written records and measures to secure the follow-up of critically ill patients over time because this was a new task that required additional knowledge for the nurses in this rural setting. Even with a relatively small population in the area, the distance and transport to a hospital had to be considered if and when a patient’s condition deteriorated. The nurse leaders negotiated for the need for two nurses on every shift to keep up with the simultaneous and complex work of admitting patients, monitoring patients, maintaining records, and processing incoming calls. Thus, the nurse leaders were able to link these processes in ways that integrated staffing, professional standards, routines and technology, and they expected this integration to result in a high quality of care. In addition, the nurse leaders worked to maintain and develop a workflow and expected this workflow to occur simultaneously.

The analysis demonstrates how the nurse leaders worked in the development and establishment of the emergency beds, which consisted of various tasks and elements. These tasks were performed simultaneously, at different times and during different stages of the process. The nurse leaders demonstrated an overview of the different tasks and elements and showed an understanding of how this diversity culminates in an established practice. This finding is in accordance with Kristiansen [36], who showed how nurses are able to handle the demands prescribed by managerial logic through sense making, establishing new organizational routines and incorporating the demands prescribed by their own professional logic as well as managerial logic. The implementation of the emergency beds was a result of the nurse leaders continuous efforts to create, maintain, and adjust the leadership required to enable a comprehensive service. For the nurse leaders, the recommendations in the report guided their implementation work. In principle, the nurses were required to adjust and adapt the recommendations to the local situation where the service was to be implemented and write applications for the financing of the implementation. This financial support included funding for their own positions as project managers. Their past experience and knowledge of health and care services in the region were important in this context. The nurse leaders used their knowledge to negotiate where the service would be established, the extent of nursing resources and expertise and the flow of information on the implementation work. Therefore, the nurses had considerable personal influence on the design of the service.

The nurse leaders initiatives and efforts in relation to development of the municipal emergency ward can be explained by the concepts “articulation work” [12] and Allen’s [13] description of health personnel’s “organizational work”. These concepts are used in various studies describing professionals’ contributions as “the act of connecting things together to allow movement” [13] and “the work that makes work work” [28]. These studies have been conducted in hospital settings. However, these terms are also relevant for analysing and describing health care work in rural settings. The nurse leaders articulation of their activities towards various actors helped integrate the acute ward into the emergency clinic. In addition, these professionals had the major responsibility of facilitating the realization of the new service and the daily emergency practices to provide satisfactory services to patients and, therefore, employed managerial logic explicitly and directly as indicated by Gadolin [37]. The nurse leaders combined their awareness of what care the patients might need with an understanding of organizational processes to undertake articulation to ensure the development of work procedures, the availability of staff and medical equipment to support emergency care nursing and collaboration with related healthcare services in the region to ensure integrated care for individual patients.

The nurse leaders experience and understanding of the relationships between the structural and professional aspects of healthcare were valuable as they developed the municipal emergency ward locally. This process involved the mobilization of several arrangements for care [13,27]. Their awareness of the organizational context, such as workflows, demand patterns, and resources availability, allowed them to negotiate rationally and strategically with GPs, emergency clinic nurses and stakeholders about how the emergency beds should be organized locally.

As described under Section 1.2, there were several reasons for the nurse leaders to be in charge of this development. Their previous experience with developing the intermunicipal emergency clinic was an important component. Additionally, the GPs were not very engaged in the development of the emergency ward even though it was being described in the political guidelines as a GP service. The nurse leaders foresaw how this could change the emergency clinic nurses’ work and sought to control this process in accordance to their professional standards. Other studies have shown similar results in which health care personnel became actively involved in managerial tasks as a strategy to increase their professional power [20,21]. Kirkpatrick [20] highlights the differences between professions such that management tasks allows doctors to maintain their professional dominance, whereas nurses have an opportunity to increase their autonomy. A relevant topic for further research could be to examine the implications of these tasks, i.e., managing service development and innovations, the relationship among professions, and which it the type of profession plays a role in who manage these tasks. Leavey [21] highlights how the non-professional aspects of managerial work are combined to maintain professional control over work tasks. Our study highlights the aspect of nursing work that equips nurses very well for taking on managerial tasks. For example, the alignment of activities and responsibilities across dimensions of time and space are integral components of nurses’ work [29]. The nurse leaders included this particular professional perspective into their effort to develop the municipal emergency ward locally. The nurse leaders continuously shifted their attention from the individual patient to the organization and combined this clinical and organizational knowledge in negotiations about how the new services should be organized. To develop a high-quality emergency bed service for patients, the nurse leaders focused on individual patients’ potential caring needs, their overall care arrangements, organizational relationships, and the necessary supporting resources. Various professionals, GPs, and nurses had different perspectives regarding the emergency beds. Here, collaboration required adjustments in practices, which led to considerable negotiation regarding previous and new forms of collaboration led by the nurse leaders [17]. Other studies have also shown how health professionals’ involvement in managerial tasks takes a negational form [1,2,8,21].

The nurse leaders role has been described in several studies as a key driver and proponent of the realization of health policy reforms [6,7]. Our analysis illustrates that the commitment of these professionals is underpinned by a fine-grained understanding of healthcare structures, local knowledge about the service, creativity and capacity available in the region, and health professional work. This study shows that nurses leading the processes of change in a rural community manage the change processes by combining the professional and organizational service domains. The nurse leaders balance their own professional ambitions for the service with legal directives, economic incentives and present budgets. Throughout the development process, the nurses carefully combine value-based and goal-based management concerns.

Nurse leaders organizational work is a necessary contribution to the local implementation of abstract and general political and management initiatives, and it ensures that emergency beds are considered comprehensive services for individual patients. However, this competency has rarely been acknowledged; thus, researchers, nurses, and policymakers must take action to promote this competency.

## 5. Conclusions

Health policy developments have changed the content of nurse leaders work and the place of complex emergency services in rural municipalities. Several studies investigating organizations and professions have examined how professional duties and tasks develop in the face of organizational and societal changes and how management and organization issues have become more important for professionals [14,15]. Consistent with our analytical framework, we argue that organizational and managerial tasks are integrated components of professional work [12,13,26]. When nurse leaders are allocated new responsibilities for service implementation and innovation in addition to their traditional leadership and management concerns, they must develop a broad understanding of the professional and organizational landscape of the services. Adequate professional competence and resources as well as economic and legal incentives and directives should be considered in relation to each other. The implementation process consists of considerations of both professional and organizational values. This distinctive clinical and organizational competency is applied to the development of a new service. We argue that such expertise equips nurse leaders particularly well to play a key role in the development and realization of healthcare services because nurse leaders facilitate services by bridging the gap between policy and practice. However, while the Norwegian government acknowledges the adaptation of national political reforms to local contexts, more research is needed to explore the particular organizational and professional possibilities and challenges related to various regions.

## References

[1] Salmela S., Eriksson K., Fagerström L. (2012). Leading change: A three-dimensional model of nurse leaders’ main tasks and roles during a change process. J. Adv. Nurs..

[2] McGivern G., Currie G., Ferlie E., Fitzgerald L., Waring J. (2015). Hybrid manager-professionals’ identity work: The maintenance and hybridization of medical professionalism in managerial contexts. Pub. Adm..

[3] Clark J. (2012). Medical leadership and engagement: No longer an optional extra. J. Health Organ. Manag..

[4] Croft C., Waldorff S.B., Pedersen A.R., Fitzgerald L., Ferlie E. (2015). A New Approach to hybrid leadership development. Managing Change: From Health Policy to Practice.

[5] Kaya N., Turan N., Aydın G.Ö. (2015). A concept analysis of innovation in nursing. Procedia Soc. Behav. Sci..

[6] Martinussen P.E., Magnussen J. (2011). Resisting market-inspired reform in healthcare: The role of professional subcultures in medicine. Soc. Sci. Med..

[7] Ploeg J., Skelly J., Rowan M., Edwards N., Davies B., Grinspun D., Bajnok I., Downey A. (2010). The role of nursing best practice champions in diffusing practice guidelines: A mixed methods study. Worldviews Evid. Based Nurs..

[8] Llewellyn S. (2001). ‘Two-way windows’: Clinicians as medical managers. Organ. Stud..

[9] Ferlie E., Fitzgerald L., Wood M., Hawkins C. (2005). The nonspread of innovations: The mediating role of professionals. Acad. Manag. J..

[10] Norwegian Ministry of Health and Care Service (2008). The Coordination Reform. Proper Treatment-at the Right Place and Right Time.

[11] Leonardsen A.-C.L., Del Busso L., Grøndahl V.A., Ghanima W., Jelsness-Jørgensen L.-P. (2016). General practitioners’ perspectives on referring patients to decentralized acute health care. Fam. Pract..

[12] Strauss A.L., Fagerhaugh S., Suczet B. (1997). Social Organization of Medical Work.

[13] Allen D. (2014). The Invisible Work of Nurses: Hospitals, Organisation and Healthcare.

[14] Noordegraaf M. (2015). Hybrid professionalism and beyond: (New) forms of public professionalism in changing organizational and societal contexts. J. Prof. Organ..

[15] Evetts J. (2009). New professionalism and new public management: Changes, continuities and consequences. Comp. Sociol..

[16] Madsen M.H., Waldorff S.B., Pedersen A.R., Fitzgerald L., Ferlie E. (2015). The role of the quality coordinator: Articulation work in quality development. Managing Change: From Health Policy to Practice.

[17] Raknes G., Hansen E.H., Hunskaar S. (2013). Distance and utilisation of out-of-hours services in a Norwegian urban/rural district: An ecological study. BMC Health Serv. Res..

[18] The Norwegian Directorate of Health (2016). Kommunenes Plikt Til Øyeblikkelig Hjelp Døgnopphold (Guidlines on Municipalities Obligation to Offer Municipal Acute Wards).

[19] Leonardsen A.-C. (2017). Experiences with Decentralized Acute Healthcare Service from Different Steakholder´s Perspective. Ph.D. Thesis.

[20] Kirkpatrick I., Dent M., Jespersen P.K. (2011). The contested terrain of hospital management: Professional projects and healthcare reforms in Denmark. Curr. Sociol..

[21] Levay C., Waks C. (2009). Professions and the pursuit of transparency in healthcare: Two cases of soft autonomy. Organ. Stud..

[22] Thorton P.H., Ocasio W., Lounsbury M. (2012). The Institutional Logics Perspektive—A New Approach to Culture.

[23] Abbott A. (1988). The System of Professions: An Essay on the Division of Expert Labor.

[24] Bévort F., Suddaby R. (2015). Scripting professional identities: How individuals make sense of contradictory institutional logics. J. Prof. Organ..

[25] Waring J., Currie G. (2009). Managing expert knowledge: Organizational challenges and managerial futures for the UK medical profession. Organ. Stud..

[26] Postma J., Oldenhof L., Putters K. (2014). Organized professionalism in healthcare: Articulation work by neighbourhood nurses. J. Prof. Organ..

[27] Allen D. (2018). Analysing healthcare coordination using translational mobilization. J. Health Organ. Manag..

[28] Suchman L., Kling R. (1996). Supporting articulation work. Computerization and Controversy, Value, Conflicts and Social Choices.

[29] Hampson I., Junor A. (2005). Invisible work, invisible skills: Interactive customer service as articulation work. New Technol. Work Employ..

[30] Chambliss D. (1996). Beyond Caring: Hospital. Nurses and the Social Organization of Ethics.

[31] Rankin J.M., Campbell M.L. (2006). Managing to Nurse: Inside Canada´s Health Care Reform.

[32] Czarniawska B. (2007). Shadowing and Other Techniques for Doing Fieldwork in Modern Societies.

[33] Smith D.E. (2006). Institutional Ethnography as Practice.

[34] King N., Cassell G.S.C. (1998). Template analysis. Qualitative Methods and Analysis in Organizational Research: A Practical Guide.

[35] Dyb K., Halford S. (2009). Placing globalizing technologies: Telemedicine and the making of difference. Sociology.

[36] Kristiansen M., Obstfelder A., Lotherington A.T. (2015). Nurses’ sensemaking of contradicting logics: An underexplored aspect of organisational work in nursing homes. Scand. J. Manag..

[37] Gadolin C. (2018). Professional employees’ strategic employment of the managerial logic in healthcare. Qual. Res. Organ. Manag. Int. J..

